# iDamIDseq and iDEAR: an improved method and computational pipeline to profile chromatin-binding proteins

**DOI:** 10.1242/dev.139261

**Published:** 2016-11-15

**Authors:** Jose Arturo Gutierrez-Triana, Juan L. Mateo, David Ibberson, Soojin Ryu, Joachim Wittbrodt

**Affiliations:** 1 Centre for Organismal Studies (COS), University of Heidelberg, Im Neuenheimer Feld 230, Heidelberg D-69120, Germany; 2 Deep Sequencing Core Facility, Cell Networks, University of Heidelberg, Im Neuenheimer 267, Heidelberg D-69120, Germany; 3 Developmental Genetics of the Nervous System, Max Planck Institute for Medical Research, Jahnstrasse 29, Heidelberg D-69120, Germany; 4 Focus Program Translational Neuroscience, University Medical Center, Johannes Gutenberg University Mainz, Langenbeckstr. 1, Mainz D-55131, Germany

**Keywords:** DamID, Transcription factor, Transcriptional regulation, Chromatin profiling, Epigenetics

## Abstract

DNA adenine methyltransferase identification (DamID) has emerged as an alternative method to profile protein-DNA interactions; however, critical issues limit its widespread applicability. Here, we present iDamIDseq, a protocol that improves specificity and sensitivity by inverting the steps *Dpn*I-*Dpn*II and adding steps that involve a phosphatase and exonuclease. To determine genome-wide protein-DNA interactions efficiently, we present the analysis tool iDEAR (iDamIDseq Enrichment Analysis with R). The combination of DamID and iDEAR permits the establishment of consistent profiles for transcription factors, even in transient assays, as we exemplify using the small teleost medaka (*Oryzias latipes*). We report that the bacterial Dam-coding sequence induces aberrant splicing when it is used with different promoters to drive tissue-specific expression. Here, we present an optimization of the sequence to avoid this problem. This and our other improvements will allow researchers to use DamID effectively in any organism, in a general or targeted manner.

## Abstract (precis)

**Summary:** Critical improvements to the DamID protocol improve specificity and sensitivity in determining genome-wide protein-DNA interactions in transient or stable transgenic animal lines.

## INTRODUCTION

Animal development is the result of an exquisite orchestration of changes in gene expression in time and space. Transcription factors (TFs) and other chromatin-associated proteins are fundamental elements in these processes and the search for their targets and the logic by which they are regulated in the genome is a central theme in today's research. Two methods are currently used to profile transcription factor-binding regions in the genome: chromatin immunoprecipitation (ChIP) and DNA adenine methyltransferase identification (DamID) (reviewed by Aughey and Southall, 2016; Furey, 2012). ChIP relies on antibody-based capture of protein-DNA complexes on crosslinked and sheared chromatin. Although this technique is solid and robust, its major drawback is its dependence on highly specific precipitating antibodies. In particular, cross-reacting antibodies may simultaneously immunoprecipitate more than one TF in a ChIP experiment. DamID offers a suitable solution to these problems. In DamID, the fusion of a TF to the bacterial gene DNA adenine methyltransferase, *Dam*, allows a restricted methylation of adenine residues of the GATC target sequences near the TF binding sites. These regions are subsequently enriched by digesting gDNA with the restriction enzyme *Dpn*I and linker-mediated PCR (LM-PCR). The PCR products are hybridized to microarrays or used directly for deep sequencing. In summary, DamID requires relatively low input material and processing time, is cost-effective and accurately reflects ChIP results (Southall et al., 2013).

DamID has been used successfully in model organisms, including *Drosophila melanogaster* (Van Steensel and Henikoff, 2000; Southall et al., 2013), *Caenorhabditis elegans* (Schuster et al., 2010), *Arabidopsis thaliana* (Germann et al., 2006) and mammalian cell cultures (Vogel et al., 2007). The current protocols require tight control to ensure low expression levels of the *E.coli* Dam methylase fused to the protein of interest. In the process of implementing DamID to developing medaka and zebrafish embryos using different transcription factors, we faced serious problems such as lack of any DamID product, non-specific amplification (*Dpn*I-independent amplification) and lack of tissue-specific expression of Dam fusion proteins.

To overcome these drawbacks and allow a wider, immediate application of the technique, we have made a series of improvements to the original iDamIDseq protocol, resulting in a method that is easily applicable and provides consistent results. This approach permits transcription factor profiling even in transient applications. We complement these experimental improvements with iDEAR (iDamID Enrichment Analysis with R), an analysis pipeline associated with iDam, as a rapid new method for establishing highly reliable profiles of transcription factor-binding sites.

## RESULTS AND DISCUSSION

### The iDamIDseq protocol: problems and solutions

**Fig. 1.**
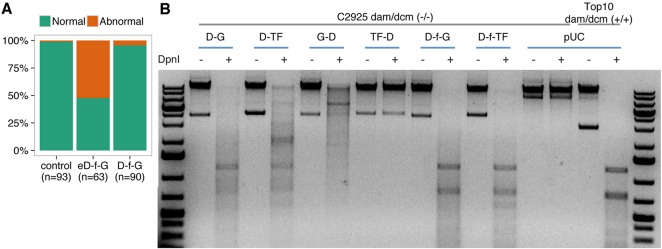
**Improving Dam-fusion proteins.** (A) DamL122A displays low toxicity in medaka embryos compared with the unmodified protein. Medaka zygotes were injected with mRNA coding for the *E. coli* Dam (eD-f-G) or DamL122A fused to GFP via flexylinker (D-f-G) (see below). Embryos were scored for abnormalities at embryonic stage 25. (B) Agarose gel of isolated bacterial gDNA samples undigested (−) or digested (+) with *DpnI*. Dam activity depends on the flexilinker, and the type and orientation of the fused proteins. Bacterial gDNA isolated from a strain deficient in the dam/dcm systems is resistant to *Dpn*I digestion. This condition can be reversed in transformed bacteria only when the fusion protein generates a functional Dam. Whereas DNA from bacteria transformed with constructs coding for fusions Dam-GFP (D-G) or Dam-TF (D-TF) (OtpA from zebrafish) can be digested by *Dpn*I, DNA from GFP-Dam (G-D) and TF-Dam (TF-D) bacteria is resistant to *Dpn*I digestion. In addition, the use of flexylinker between Dam and the fusion protein (D-f-GFP and D-f-TF) generates a *Dpn*I digestion pattern similar to that of bacteria with a functional dam/dcm system (Top10 cells).

*E. coli* Dam (eDam) displays specific methylation activity on its cognate GATC but also minor unspecific methylation on near-cognate sequences (Horton et al., 2005). This means that its expression may trigger unwanted toxic effects. When mRNA coding for the fusion eDam-GFP (eD-f-G) was injected into zygotes, we observed a high number of abnormal embryos at stage 25, 52%, compared with 1% in the control case (Iwamatsu, 2004) (Fig. 1A). To overcome this problem, we used the mutant version DamL122A (Horton et al., 2005) (henceforth referred to as Dam), the activity of which has been shown to increase the specificity of methylation on GATC sites. Interestingly, injecting mRNA coding for the fusion Dam-GFP (D-f-G) produced a much lower number of abnormal embryos, 4%, which was similar to the control (Fig. 1A).

Chimeric fusion may compromise the normal functions of a protein due to steric hindrance (Arai et al., 2001). We included a flexible linker between the Dam protein and the transcription factor, and tested different orientations. We observed that the methylation defect of *Dam*-deficient bacteria could be rescued differentially by the Dam fusions depending on the orientation of the fused protein (N or C terminal), but the presence of the flexilinker always improves the activity (Fig. 1B and Fig. S1). Accordingly, all chimeric Dam constructs used for the rest of this work carry the flexilinker, indicated by the letter ‘f’ in the name of the fusions.

**Fig. 2.**
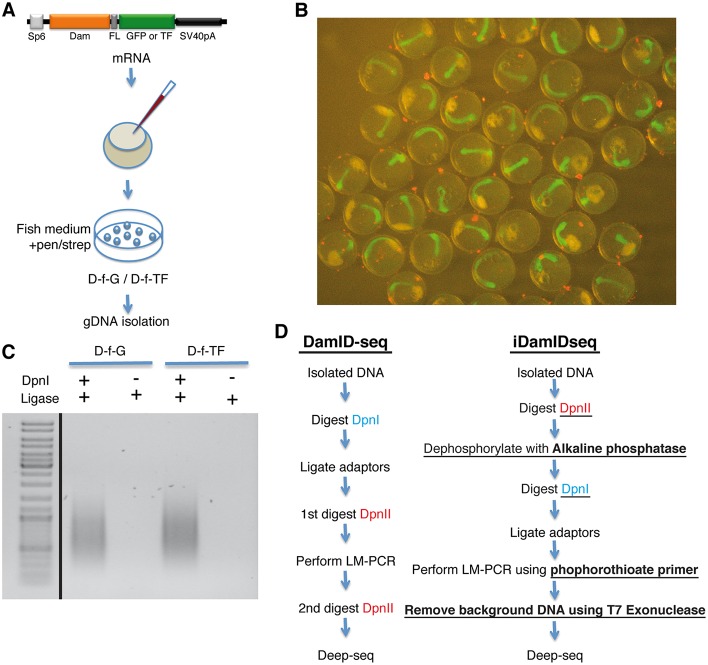
**Modification of the crucial steps of the DamID protocol.** (A) Medaka zygotes were injected with mRNA coding for Dam-f-GFP or Dam-f-TF (Medaka Rx2). Embryos were maintained in ERM supplemented with an antibiotic solution and gDNA was isolated at stage 22. (B) Medaka embryos (stage 22) expressing Dam-f-GFP. (C) DamID LM-PCR at 25 cycles using the modifications presented in the main text generates only *Dpn*I-dependent amplification (see Materials and Methods, iDamIDseq protocol). (D) Flowchart comparing the standard DamID-seq protocol (based on Wu et al., 2016) with the iDamIDseq protocol (improvements are underlined).

To address a possible impact of the Dam fusion protein on development, we injected mRNA coding for a nuclear-localized Dam-f-GFP or Dam-f-TF into medaka zygotes and allowed them to develop at 28°C (Fig. 2A). At stage 22 (Iwamatsu, 2004), embryos did not show any evident abnormality and Dam-f-GFP-injected embryos ubiquitously expressed GFP (Fig. 2B).

As we repeatedly obtained linker-mediated amplification (LM-PCR) independently of *Dpn*I (data not shown), we reasoned that this problem is due to the ligation of the adaptors to free phosphorylated 5′ ends, a result of the original genomic DNA preparation rather than *Dpn*I digestion. We enhanced the specificity of the adaptor ligation by switching the order of the *Dpn*I and *Dpn*II digestions, and by adding an alkaline phosphatase step. First, we reduced size complexity by digesting the DNA with *Dpn*II, which cuts GATC sites but is sensitive to adenine methylation. Then we treated these fragments with alkaline phosphatase and proceeded with digestion using *Dpn*I, which only cuts methylated adenine GATC sites. LM-PCR amplification products were obtained only in samples treated with *Dpn*I (Fig. 2C).

In order to prepare the sample for deep sequencing, any contaminating genomic DNA must be removed. We performed LM-PCR using primers protected with phosphorothioate modifications and then treated the samples with T7 exonuclease (Fig. 2D).

**Fig. 3.**
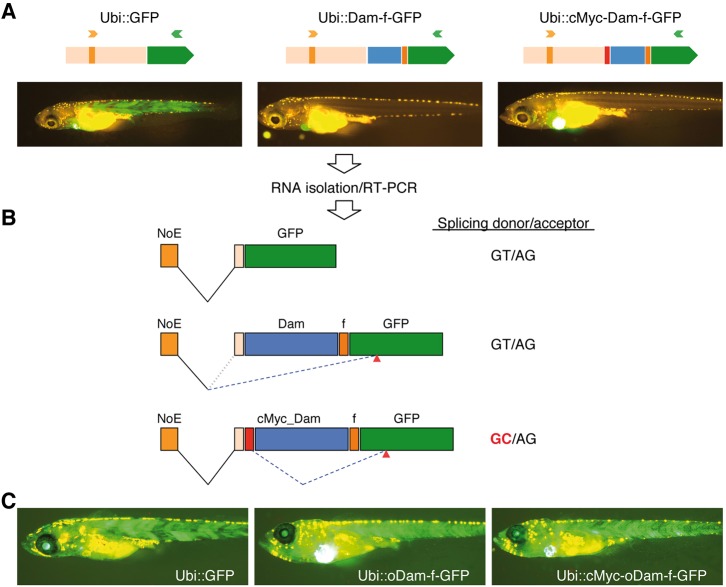
**Optimization of the bacterial *Dam* gene is necessary for proper expression of Dam fusion proteins to avoid aberrant splicing.** (A) Plasmids containing GFP, Dam-f-GFP and cMyc-Dam-f-GFP cassettes driven by the 3.5 kb ubiquitin promoter (Ubi) were co-injected with Tol2 transposase into medaka zygotes. Successfully injected larvae expressing EGFP in the heart were selected for further studies. Only Ubi::GFP is expressed ubiquitously in the body of the larvae. (B) RNA was isolated from pools of larvae from the experimental groups. RT-PCR was performed using a forward primer (orange arrowhead in A) annealing in the non-coding exon included in the ubiquitin promoter (NoE) and the reverse primer (green arrowhead in A) in the body of the GFP-coding sequence. Proper splicing occurs between NoE and GFP in the Ubi::GFP larvae. In Ubi::Dam-f-GFP larvae, incorrect splicing occurs between the NoE and a cryptic acceptor site in the GFP-coding region (red arrowheads). In the Ubi::cMyc-Dam-f-GFP, NoE is spliced to the proper acceptor upstream of the cMyc sequence, but after that the cMyc sequence is aberrantly spliced, using a cryptic donor site, to the same cryptic acceptor sequence in GFP as for Ubi::Dam-f-GFP (see also Fig. S2). The prokaryotic Dam ORF carries a strong splicing enhancer recognized in the eukaryotic context. (C) Optimization of the Dam ORF removed this potential, facilitating proper expression of the fusion proteins.

The final goal of DamID is to use specific promoters to generate transcription factor-binding profiles in a tissue-specific manner. We cloned Dam-f-GFP using promoters that included ubiquitin (Mosimann et al., 2011), heat shock (Blechinger et al., 2002) and Rx2 (Reinhardt et al., 2015) in plasmids carrying transgenesis markers such as Cmlc2:GFP or RFP. Surprisingly, none of the Dam-f-GFP constructs showed GFP expression, whereas the unfused GFP construct did (Fig. 3A; data not shown). As the Dam-f-GFP fusion itself can be translated efficiently (see Fig. 2B), we suspected problems at the transcriptional/splicing level. RT-PCR of samples from the different ubiquitin-driven constructs revealed the aberrant splicing of the *Dam* gene out of the final transcript (Fig. 3A,B; Fig. S2). A customized optimization of the *Dam* gene (*oDam*) removed the cryptic splicing regulatory sites and restored the expression of the GFP in the larvae (Fig. 3C; Fig. S3).

### Proof of concept validation and data analysis with iDEAR

As a proof of concept, and to reveal the specific enrichment of transcription factor DamID products, we applied this technique to medaka using the transient expression of the transcription factor Rx2, which is the homolog of the mammalian Rax homeodomain proteins involved in retina development. We injected mRNA coding for a nuclear localized Dam-f-GFP or Dam-f-Rx2, extracted gDNA and processed the samples as described above with two biological replicates per condition.

**Fig. 4.**
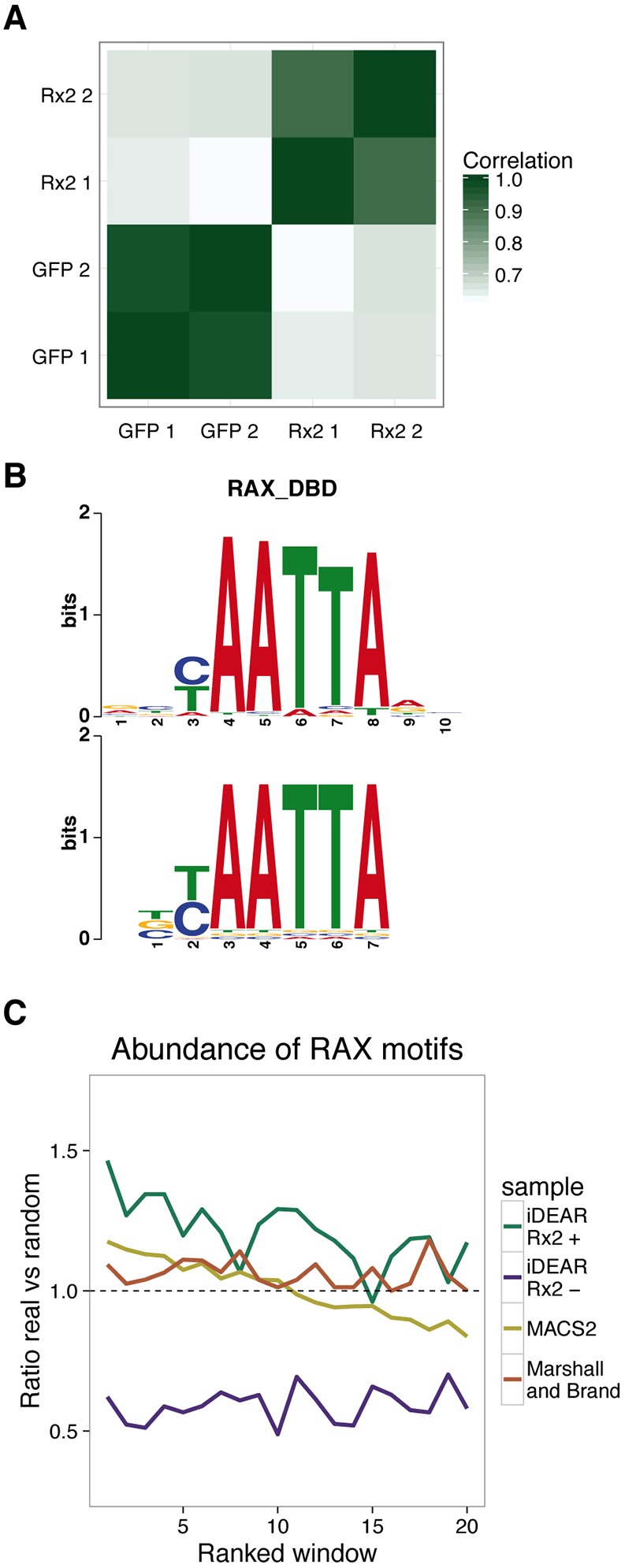
**Analysis of iDamIDseq results on Rx2.** (A) Samples showed high correlations between replicates and low correlations between Rx2 and GFP, based on the genome-wide read coverage. (B) The most overrepresented motif found *de novo* has a consensus sequence BYAATTA, very similar to the binding motif known for the mammalian Rax protein. (C) Abundance of the RAX motif in the identified sites, with respect to random sequence, correlates with the score of these regions. The Rx2-enriched sites identified by iDEAR show a higher enrichment than those represented by MACS2 and the Marshall and Brand pipeline.

The correlation of read coverage over the genome is very high between replicates but quite distinct between Rx2 and GFP, showing the consistency and specificity of this method (Fig. 4A). We developed an R package, named iDEAR (iDamID Enrichment Analysis with R, available at https://bitbucket.org/juanlmateo/idear), to facilitate the straightforward analysis of regions that undergo differential methylation (see Materials and Methods). Using iDEAR, we were able to identify 7948 Rx2 target regions (Table S1). Strikingly, we also identified 6255 regions with a significant depletion of the signal in the Rx2 samples compared with GFP. Based on the distance to the closest transcription start site (TSS, Fig. S4A,B), such Rx2-occupied sites tend to be within 10 kb and 50 kb of genes, reflecting enhancers, whereas Rx2-negative sites are mostly in the close vicinity of a TSS, showing a profile similar to promoters. We concluded that Rx2-depleted sites predominantly correspond to promoters of actively transcribed genes that are situated within regions of very accessible chromatin but are not bound by Rx2.

Using DREME (Bailey, 2011) as a *de novo* motif discovery tool to compare Rx2-occupied versus Rx2-negative sites, the top hit was the motif BYAATTA, which is almost identical to the motif identified *in vitro* by SELEX for the mammalian Rax protein (Jolma et al., 2013) (Fig. 4B). This indicates that Dam-f-Rx2 shows specific binding that recognizes the motif demonstrated for its human ortholog, even in overexpression conditions.

To evaluate the performance of iDEAR, we compared it with other tools used for similar purposes: MACS2 (Zhang et al., 2008) and the pipeline proposed by Marshall and Brand (2015). MACS2 produced 40,292 peaks and Marshall and Brand only 1635 sites. Although the number of identified sites by MACS2 is very different from the number of sites identified by iDEAR, their average length is very similar at around 1 kb; but Marshall and Brand's pipeline produced extremely large sites of enrichment (Fig. S4C).

Knowing that the RAX motif is the most over-represented motif in the Rx2 sites, we wanted to see whether its presence correlates with the score that each tool assigns to the sites. To check this, we computed the ratio of sites with the motif in their sequence versus random sequences ordered by score. Rx2-occupied sites identified by iDEAR showed a higher ratio than the other tools (always greater than 1) and correlated well with the score, i.e. a higher abundance of motifs was found in sites ranked higher (Fig. 4C). We found the same correlation for MACS2, but half of the peaks this method identified have a lower content of the motif than expected at random. This finding may indicate a high false-positive rate in peak calling, which is also expected by the very large number of peaks that MACS2 finds. We need to note that MACS2 was designed to analyze ChIP-seq data where the enrichment of a TF is clearly identified by the so-called ‘peak’. The fact that iDamIDseq data does not necessarily show a clear and single peak per bound region, in addition to the inability of MACS2 to handle replicates, explains the lower performance of this tool with respect to iDEAR.

In order to gain insights into the potential functional properties of the sites identified by iDEAR, we looked into their overlap with regions constrained by evolution. The Rx2 sites identified by iDEAR overlapped to a higher degree with conserved sites in fish than the sites identified by the other two tools (Fig. S4D).

Although Rx2 was provided as mRNA and was therefore expressed in the whole embryo, a careful inspection of the regions of Rx2 enrichment revealed many players known to be involved in retinal development, including Six3 (Loosli et al., 1998), Otx2 (Zuber et al., 2003), Pax6 (Loosli et al., 1998) and Sox2 (Reinhardt et al., 2015). Interestingly, we also found enrichment of Rx2 on its own proximal upstream locus (Fig. S5A). We generated a reporter element with the sequence of the Rx2 enriched region in front of a minimal promoter and GFP. This element drives GFP expression in the photoreceptor cell layer and overlaps completely with the Rx2 expression domain in these cells (Fig. S5B). Future analysis will require a fusion protein specifically expressed in the Rx2 expression domain in the retina.

In conclusion, the improvements described above to the DamID protocol preserve the full chromatin profiling capacity of the ‘classical’ technique, but substantially reduce unwanted background noise and consequently increase sensitivity and specificity. iDamIDseq can be readily applied to determine transcription factor-binding profiles even in transient assays. Our optimization of the Dam-coding sequence facilitates proper tissue-specific expression, making it compatible with any organism that is amenable to transient or stable transgenesis.

## MATERIALS AND METHODS

### Fish maintenance

Medaka (*Oryzias latipes*) fish were bred and maintained as previously established (Loosli et al., 2000). The animals used in the present study were from the inbred strain Cab. All experimental procedures were performed according to the guidelines of the German animal welfare law and approved by the local government (Tierschutzgesetz §11, Abs. 1, Nr. 1, husbandry permit number 35–9185.64/BH Wittbrodt).

### Plasmids

The variant DamL122A was created by site-directed mutagenesis of the *E. coli Dam* gene using mutagenesis primers and flanking primers (Heckman and Pease, 2007). (All primers used in this work are listed in Table S1.) The flexible linker was cloned as a dsOligo that encodes four GGGS amino acid repeats. The repeat sequences are flanked by *Nhe*I and *Spe*I sites. The mmGFP was amplified from plasmid pT2-*otp*ECR6_E1B::mmGFP (monomeric GFP, see Gutierrez-Triana et al., 2014). All fragments were cloned into the pCS2+ vector (Rupp et al., 1994) as either N- or C-terminal fusions, followed by the SV40_polyadenylation signal of the pCS2 vector. Plasmid integrity was confirmed by sequencing.

We used the gene synthesis service of GeneArt (Thermo Fisher Scientific) to obtain the optimized Dam sequence (oDam). In addition to codon optimization, cryptic splice sites were avoided (the DNA sequence is shown as an alignment to the unmodified DamL122A in Fig. S3). We replaced the DamL122A with the optimized *Dam* in the pCS2+ plasmids described above.

The DamL122A or oDam cassettes were excised from the pCS2+ plasmids using *Age*I and *Not*I, and subcloned downstream of the 3.5 kb zebrafish *ubiquitin* promoter (Mosimann et al., 2011) in a Tol2_based plasmid (Kawakami et al., 1998), with *cmlc2*::EGFP as the insertional reporter (Rembold et al., 2006). The Rx2 DamID-enriched region (Rx2_DBS) was amplified from medaka genomic DNA and the fragment was used to replace the otpECR6 element of the pT2-*otp*ECR6_E1B::mmGFP plasmid mentioned above using *Asc*I-*Spe*I.

### Microinjection

mRNA was synthesized using mMesage_mMachine SP6 kit (Thermo Fisher Scientific, AM1340) on linearized pCS2+ templates. Medaka zygotes were injected with mRNA at 10 ng/µl. The progenies of injected fish were maintained in ERM medium (Loosli et al., 2000) supplemented with penicillin_streptomycin (P0781, Sigma-Aldrich, 200 units/200 µg per ml of ERM). Embryos were collected at stage 22 (34 hpf at 28°C). Unfertilized and dead embryos were removed. Recombinant ubiquitin promoter plasmids and the pT2-Rx2_DBS::mmGFP plasmid were injected into medaka zygotes at 10 ng/µl in the presence of 10 ng/µl Tol2 transposase mRNA. The injected embryos were maintained in ERM medium supplemented with 0.2 mM N-phenylthiourea (Sigma-Aldrich, P7629) until hatching. pT2-Rx2_DBS::mmGFP-injected embryos were screened for RFP+ hearts and raised to generate the transgenic line Tg(Rx2_DBS::GFP).

### *Dpn*I protection assay

pCS2+ plasmids carrying the cassettes coding for every particular *Dam* fusion protein were used to transform the *E. coli* strain C2925, deficient in the dam/dcm methylation system. As control, the pUC19 plasmid was used to transform C2925 cells and One Shot TOP10 cells, which have a normal methylation system. Bacterial genomic DNA was isolated from 3 ml LBamp cultures from individual colonies using the DNeasy Tissue kit (Qiagen, 69504). gDNA (1 µg) was digested with 10 units of *Dpn*I (NEB, R0176S) for 1 h at 37°C. The products were run in a 1% agarose gel.

### iDamIDseq protocol

#### gDNA isolation

Embryos (20-30) or tissue were washed with 1× ERM or 1× PBS, respectively, removing as much media as possible and homogenized using a pestle in 400 µl of TEN buffer [100 mM Tris-HCl (pH 8.5), 10 mM EDTA, 200 mM NaCl, 1% SDS] plus 20 µl of 20 mg/ml Proteinase K. Samples were incubated overnight at 50-60°C then cooled down to room temperature for 5 min. RNase A (20 µl of 10 mg/ml; DNase and Proteinase-free, Thermo Fisher Scientific, EN0531) was added then samples were incubated for 15 min at room temperature. Phenol:chloroform:isoamylalcohol (25:24:1, 600 µl) (Roth, A156.1) was added and mixed by inversion. Samples were then incubated for 10 min at room temperature then centrifuged at 10,000 rpm at room temperature for 20 min. The aqueous phase was transferred to a tube containing 600 µl of chloroform, mixed and centrifuged at 10,000 rpm for 10 min. The resultant aqueous phase was transferred into a tube containing 600 µl of isopropanol, mixed, stored at −20°C for 30 min then centrifuged at 10,000 ***g*** at 4°C for 20 min. The supernatant was removed and added to 800 µl of ice-cold 70% ethanol, then centrifuged at 20,000 ***g*** at room temperature for 10 min. As much supernatant as possible was removed and the pellet was dried at 60°C for 10 min before adding 50 µl of pre-warmed water (60°C). Tubes were incubated for 10-20 min at 60°C, with gentle flicking of the tube sporadically until the pellet was dissolved. Quality was checked by measuring OD_260_ (above 1.80) and gel electrophoresis.

#### *Dpn*II digestion and alkaline phosphatase treatment

In a 20 µl reaction, 2 µl 10× NEB3.1 buffer, 1 µg of gDNA and 10 units of *Dpn*II (NEB, R0543S) were mixed and incubated for 6 h at 37°C. The enzyme was inactivated by incubation at 65°C for 20 min. To the inactive *Dpn*II reaction, 23 µl H_2_O, 5 µl 10× AP buffer and 5 units of antarctic phosphatase (NEB, M0289) were added, then the mixture was incubated for 1 h at 37°C and inactivated at 70°C for 10 min. The reaction was cleaned up using an InnuPREP Double EPure Kit (Analytik Jena) and eluted in 12 µl of H_2_O.

#### *Dpn*I digestion

In a 10 µl reaction, 1 µl CutSmart buffer, 5 µl of *Dpn*II/AP-treated sample and 10 units of *Dpn*I enzyme (NEB, R0176S) were mixed. *Dpn*I was excluded from the control sample. The reaction was incubated at 37°C for 12 h then inactivated at 80°C for 20 min.

#### Adaptor ligation

The treated samples (±*Dpn*I) were added to 2 µl 10× T4 ligase buffer, 1 µl of 50 µM dsOligos AdRt/AdRb, 2.5 units of T4 DNA ligase (Thermo Fisher Scientific, EL0011) and 6.5 µl H_2_O, and incubated overnight at 16-18°C. The reaction was cleaned up using an InnuPREP Double EPure Kit (Analytik Jena) and eluted in 50 µl of H_2_O.

#### LM-PCR

ThermoPol Buffer (10×, 2.5 µl), 1 µl 10 mM dNTPS, 1 µl 10 µM AdR_PCR primer (Table S1), 5 µl of ligation sample and 1.25 units of Taq polymerase (NEB, M0267S) were added to a 25 µl reaction. PCR was carried out as follows: 68°C for 10 min; 1 cycle of 94°C for 15 s, 65°C for 30 s and 68°C for 5 min; and 20-30 cycles of 94°C for 15 s, 65°C for 30 s and 68°C for 2 min. The exact number of cycles was determined experimentally. Each PCR sample (5 µl) was run on a 1% agarose gel to confirm the presence of a smear in the *Dpn*I+ samples (around 200 bp to 2 kb).

#### T7 exonuclease treatment

CutSmart buffer (5 µl, 10×) , 10 units T7 exonuclease (NEB, M0263S) and 14 µl H_2_O were added to 30 µl of clean LM-PCR. The sample was incubated for 1 h at 25°C, cleaned up using an InnuPREP Double EPure Kit (Analytik Jena) and eluted in 20 µl H_2_O ready for library preparation. In order to obtain the minimum amount of DNA for deep sequencing it was sometimes necessary to repeat the PCR step and pool the DNA samples.

### Sequencing

DNA samples were fragmented using the Covaris S2 sonicator in AFA microtubes. The library was then prepared using the NEBNext Ultra DNA Library Prep kit for Illumina (E7370, NEB) with NEBNExt Multiplex Oligos for Illumina (E7500, NEB). Sequencing was performed with the Illumina HiSeq 2500 sequencing system.

### Sequencing data processing

Reads were mapped to the medaka genome (Kasahara et al., 2007) (oryLat2 assembly) using Bowtie2 (Langmead and Salzberg, 2012) with default parameters. The mapped reads were then filtered with SAMtools (Li et al., 2009) to keep only those with a minimum mapping quality of 20.

### Identification of enriched regions

First, the set of potential *Dpn*I fragments was built from a BSgenome object for the oryLat2 assembly using the function vMatchPattern with the restriction site ‘GATC’. Only fragments spanning adjacent predicted restriction sites with lengths ranging from 200 to 2000 bases were considered. Next, the reads that fell into each predicted fragment were counted for each of the samples, with the function summarizeOverlaps, setting the parameter ignore.strand to TRUE. These counts were used to produce the correlation heatmap in Fig. 4A. In order to discard fragments with spurious mapped reads, only fragments with a minimum number of reads relative to the fragment length were kept. This threshold was computed as three times the total number of reads in all fragments that were considered, divided by their total length. After this selection, fragments that were not further apart than the smallest fragment length were joined together. With the resulting set of genomic regions, the read count was computed again with summarizeOverlaps and the resulting matrix was used to compute significant differences between samples using DESeq2 (Love et al., 2014). The R package iDEAR implements this data analysis pipeline and is available at https://bitbucket.org/juanlmateo/idear.

### *De novo* motif discovery

DREME (Bailey, 2011) was used to search for the most enriched motifs in the sequence within the coordinates of the Rx2-positive sites (parameter –p), compared with the sequence within the coordinates of the Rx2-negative sites (parameter –n).

### Motif enrichment

FIMO (Grant et al., 2011) was used to identify motif matches of the RAX-binding motif (Jolma et al., 2013) (RAX_DBD) in the sequence within the coordinates of each region that had been identified. All the regions from each set were sorted by significance (from highest to lowest) and divided in 50 bins. For each bin, a ratio was computed as the number of original sequences with at least one motif match divided by the number of shuffled sequences with at least one motif. The shuffled sequences were generated by randomly permuting dinucleotides for each individual sequence that was analyzed.

### Association of sites to genes

Each site identified by iDEAR was associated with the gene whose transcription start site is closest, or overlapping, on either side of it. Version 84 of medaka transcripts in ENSEMBL was used for this.

### Analysis with MACS2 and the Marshall and Brand pipeline

For comparison, the mapped reads were also subjected to analysis with other tools, including MACS2 (Zhang et al., 2008) and the method proposed by Marshall and Brand (2015). MACS2 was invoked with the parameters –broad-cutoff 0.01 –broad –nomodel –extsize 300 –gsize 7e8 and –t, which provided the bam files of the two Rx2 replicates, and –c, which provided the bam files for the two GFP replicates. For the other tool, the script damidseq_pipeline was invoked, providing the bam files of the two Rx2 replicates and, as this script cannot handle replicates as controls, only the first replicate of GFP was provided with the parameter --dam. In this case we used the same coordinates for GATC fragments as we had with iDEAR, using the parameter --gatc_frag_file. After this, the script find_peaks was used over the bedgraph output produced by the previous script.

### Conservation analysis

The phastCons 5-Way track for medaka was downloaded through the Table Browser (Karolchik, 2004) from the UCSC Genome Browser as a bed file. For each enriched site identified as an Rx2-binding site by iDEAR, MACS2 or the Marshall and Brand tool, the proportion of bases in the site that are also covered by a phastCons element were also computed.

### RT-PCR

Ten hatchling larvae (10 dpf) per experimental group, expressing EGFP in the heart, were transferred to a 1.5 ml tube and total RNA was isolated using Trizol reagent (Thermo Fisher Scientific, 15596026), according to the manufacturer's instructions. DNase-treated total RNA (1 µg) was used to synthesize cDNA. PCR was carried out with Q5 polymerase (NEB, M0491) using 1× Q5 buffer, 200 µM dNTPs, 0.5 µM RT_Ubi fwd primer, 0.5 µM RT_mmGFP rev primer (Table S1), 2 µl of cDNA and 0.02 units/µl Q5 polymerase. Cycling parameters were: 94°C for 2 min; 35 cycles of 94°C for 15 s, 60°C for 30 s and 72°C for 1 min; 72°C for 5 min. PCR products were analyzed in an agarose gel and sequenced.

### Immunohistochemistry

Immunostainings were performed on 16 μm cryosections as previously described (Inoue and Wittbrodt, 2011) using the primary antibodies rabbit anti-OlRx2 (1:250; Reinhardt et al., 2015) and chicken anti-GFP (1:500, A10262, Thermo Fisher Scientific), and the secondary antibodies goat anti-rabbit IgG Alexa Fluor 647 (1:1000, A-21245, Thermo Fisher Scientific) and goat anti-chicken IgY Alexa Fluor 488 (1:1000, A-11039, Thermo Fisher Scientific).
